# Diminished general and sexual hedonic capacity in schizophrenia - a clinical and neural characterization

**DOI:** 10.3389/fpsyt.2026.1824523

**Published:** 2026-08-14

**Authors:** S. Hamers, C. Sinke, N. Schultz, T.H.C. Krüger

**Affiliations:** 1 Division of Clinical Psychology and Sexual Medicine, Department of Psychiatry, Social Psychiatry and Psychotherapy, Hannover Medical School, Hannover, Germany; 2 Center for System Neuroscience, Hannover Graduate School for Neurosciences, Infection Medicine and Veterinary Sciences of the University of Veterinary Medicine, Hannover, Germany

**Keywords:** anhedonia, fMRI, reward processing, schizophrenia, sexual dysfunction

## Abstract

**Background:**

Individuals with Schizophrenia are often reported to suffer from sexual dysfunction, but despite its clinical and individual relevance, the underlying neural mechanisms have not been investigated. To date it is unclear whether sexual problems are inherent to the disease, a secondary symptom, or a side effect of the antipsychotic agents. The present study aimed to investigate issues with sexual functioning in schizophrenia in relation to negative symptoms and anhedonia on a clinical and neural level.

**Methods:**

29 people with Schizophrenia and 29 healthy controls were assessed for schizophrenia-specific symptoms, anhedonia and problems in sexual functioning. The SEXpect paradigm, imaging anticipatory and consummatory responses to sexual images, was applied in an MRI scanner to investigate group differences in sexual reward processing and clinical correlates of potentially altered functional brain response.

**Results:**

Healthy individuals showed higher activations during sexual anticipation in the precuneus and cerebellar, occipital, and paracentral regions compared to people with schizophrenia. During image consumption, controls showed increased response in the calcarine gyrus. Functional brain response in all relevant clusters was associated with negative symptoms and sexual functioning scores. Further, clinical scales for negative symptoms, anhedonia and sexual functioning showed strong correlations with each other and were not affected by medication parameters.

**Conclusion:**

Present results suggest that deficits in sexual functioning in schizophrenia are not merely due to antipsychotic medication but could be related to or share common mechanisms with negative symptoms and anhedonia.

## 1 Introduction

Schizophrenia (SZ), estimated to affect about 1% of the world’s population, is characterized by positive (e.g., hallucinations, delusions) and negative (e.g., avolition, flat affect) symptoms and cognitive deficits (e.g., in working memory (WM)) (1). Commonly, SZ is treated with antipsychotic medication, which improves positive symptoms for most patients. However, these agents often do not sufficiently relieve negative symptoms and can come with significant side effects, impacting the patient’s quality of life immensely and long-term (2, 3).

An aspect of SZ, which has only recently started to receive attention, is sexual dysfunction (SD). Prevalence estimates suggest up to 96% of patients to experience sexual issues, including loss of libido, orgasm deficits, and erectile/lubrication issues (4). SD adversely affects patients’ well-being and quality of life and poses a significant risk to treatment adherence due to it often being dismissed as a medication side effect, contributing to treatment discontinuation (5). On the one hand, antipsychotic medication does target the dopaminergic system, potentially leading to increased prolactin levels, acknowledged to underlie side effects, including SD. In that respect, first-generation antipsychotics (FGA) are associated with higher side effect frequency and severity than second-generation antipsychotics (SGA) (6, 7). On the other hand, several recent studies indicate that SD is not due to medication alone but seems to be inherent to the disease, as it has been found in unmedicated patients, independent of type of medication, and has been associated with negative symptoms (5, 8). Currently, the aetiology of SD in SZ is unknown, but several explanations are possible. Besides being a medication side effect, it could be a primary symptom of the disease or a secondary symptom caused by difficulties entering and maintaining romantic relationships due to symptoms and stigmatization (5). We want to propose SD to be potentially related to SZ itself and to be linked to the negative symptom dimension of the disorder, specifically anhedonia. Anhedonia describes the reduced capacity to experience pleasure and is a core negative symptom of SZ. Currently, the literature supports the ‘liking-wanting paradox’, describing consummatory pleasure in SZ to be largely intact but the anticipatory response to be disrupted (9–11). The exact neurocognitive processes underlying the phenomenon remain unclear, but likely contenders are a faulty value representation and disrupted reward learning.

The search for neural mechanisms underlying SZ has given rise to multiple theoretical models. Among the most prominent and widely accepted is the dopamine hypothesis. This theory is supported by observations that dopamine receptor blockade - the principal mechanism of action of antipsychotic agents - alleviates positive symptoms, whereas increased dopamine levels, e.g., through amphetamines, induces psychosis-like symptoms (1). Positive symptoms are therefore linked to increased dopamine levels in limbic regions and mesolimbic pathways, while negative symptoms are thought to originate in decreased dopaminergic activity in frontal cortical areas and mesocortical pathways (2). SZ has been associated with multiple structural alterations, including ventricular enlargement and widespread volume reductions in frontal and temporal regions and reductions in cerebral blood flow (1, 12).

Negative symptoms, especially the anhedonia-amotivation dimension, are usually linked to deficits in reward processing. Aberrant reward ‘wanting’ (anticipation) is consistently associated with reduced functional activation in the ventral striatum (VS), alongside hypoactivations in the insula, ventromedial prefrontal cortex, and anterior cingulate cortex (ACC) (10, 13). Findings on reward ‘liking’ (consummation) remain mixed, with reports of intact, hyper- and hypoactivity of the VS and abnormalities in the insula, orbitofrontal cortex (OFC), ACC, prefrontal cortex, and amygdala (10, 13). Lastly, deficits in reward valuation and positive reinforcement learning have been connected to hypoactivations in, e.g., the VS, frontal regions, the precentral gyrus, and cerebellar regions (3, 10, 13, 14).

Despite its clinical relevance, there are, to our knowledge, no studies investigating the neural correlates of SD in SZ. Nonetheless, prior work identified substantial overlap between neural mechanisms underlying both sexual processing and the emergence of SZ symptoms, specifically anhedonia, including frontal areas, the hippocampus, VS, amygdala, insula, ACC, thalamus, and ventral tegmental area (VTA) (8, 15). This convergence suggests that sexual problems may be closely linked to the negative symptomatology in SZ and may share an origin with the phenomenon of anhedonia.

The present study examined the neural activations during anticipation and consummation of sexual stimuli in SZ. We hypothesized that (1) individuals with SZ would report lower sexual functioning and hedonic capacity than healthy counterparts, (2) SZ would display hypoactivations during sexual reward processing, and (3) measures of anhedonia and SD would be associated with each other and with neural activity during sexual reward processing.

## 2 Materials and methods

### 2.1 Participants

Participants were recruited via internet and flyers. Patients were additionally recruited via in- and out-patient treatment in Hannover Medical School (MHH). Participants had to be at least 18 years old and able to undergo an MRI scan. The patient group (SZG) needed an official F20 diagnosis with no other acute psychiatric disorder and no acute psychotic episode. Healthy control participants (HC) were matched on age, gender, handedness and level of education and were excluded if acute psychiatric or neurological conditions were present. In total, 93 participants were recruited for the study and 29 per group were used in the final sample **Appendix A1**. All participants gave informed consent to participate and anonymity was ensured. Participation was reimbursed with 90€ and any money won during the study (see Behavioral Test). Data collection took place between June 2023 and March 2025 at MHH. Study design was approved by the ethics committee of MHH (No. 10568_BO_S_2022).

### 2.2 Clinical assessment

The study included an interview for sociodemographic information, a diagnostic tool for SZ (63) [Positive and Negative Syndrome Scale (PANSS)], self-assessment questionnaires for anhedonia [Dimensional Anhedonia Rating Scale 26 (DARS)] and sexual dysfunction [Arizona Sexual Experience Scale (ASEX), Change in Sexual Functioning Questionnaire (CSFQ)], and a computer test assessing effort-based decision making as a proxy for anhedonia (Effort Expenditure for Reward Task – EEfRT). In SZG, medication parameters were assessed with a focus on cumulative time taking antipsychotic medication, current chlorpromazine dose equivalence (CPZ dose), and the prolactinergic potential of the current medication (see **Appendix A2**).

#### 2.2.1Self-report questionnaires

DARS (16): Assesses anhedonia considering individual interests and different aspects of reward (interest, motivation, effort and pleasure). The DARS has 26 items, rated on a 0–4 scale with higher scores indicating greater hedonic capacity. It yields a total score and four subscales for different reward types (see **Appendix A3**).

ASEX (17): Assesses sexual functioning (SF) on five items, rated on a 1–6 scale with higher scores indicating worse sexual functioning. It yields a total score of max. 30 points and SD is indicated at more than 19 points total, if at least one item is rated with 5 or 6, or if at least three items are rated with 4 or higher.

CSFQ (18): Assesses SF on 14 items, rated on a 1–5 scale. It yields a total score and five subscores (see **Appendix A3**). Higher scores indicate better functioning and scores below 42 (females) or below 48 (males) indicate SD.

#### 2.2.2 Behavioral test

**Figure 1:**
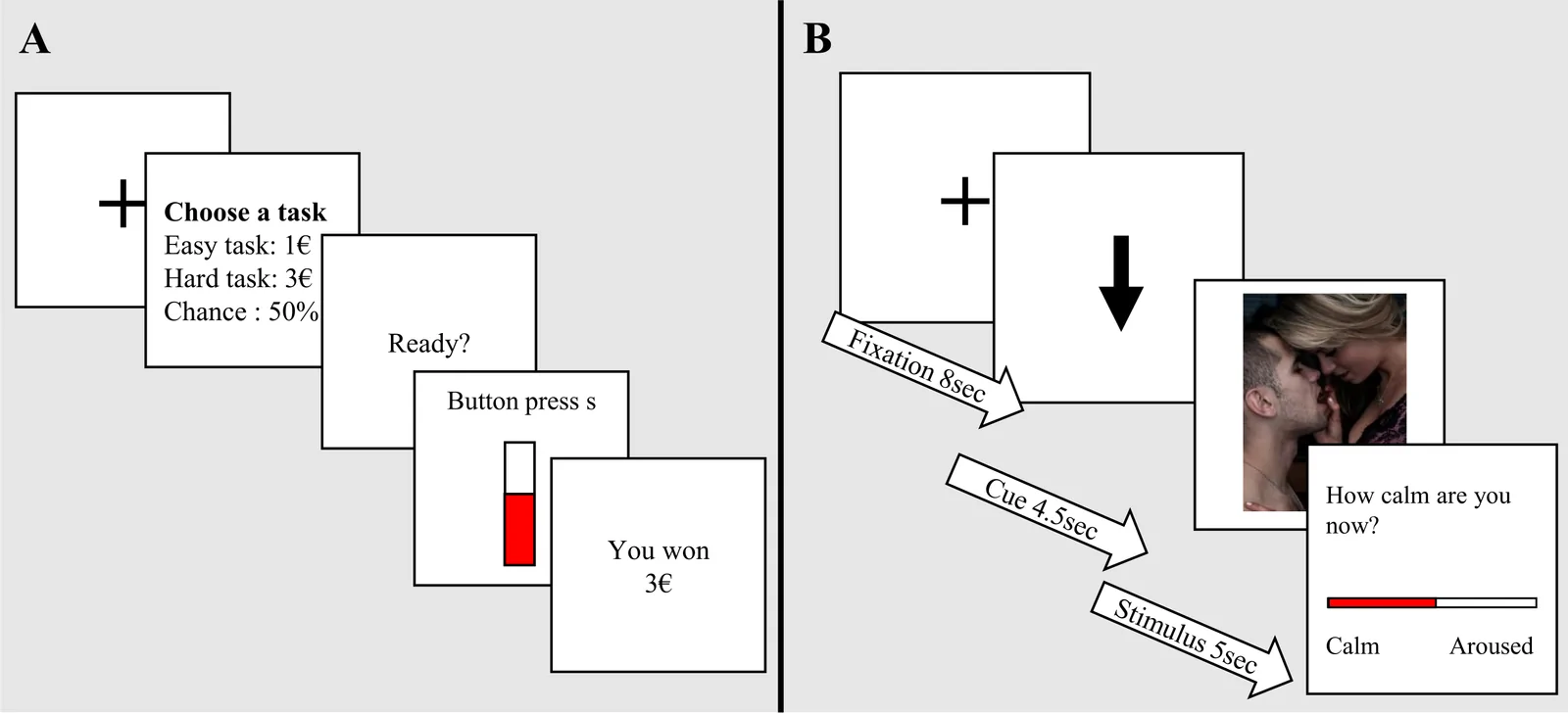
Scheme of **(A)** EEfRT and **(B)** SEXpect paradigm. Source: Photograph from © Nencki Affective Picture System (NAPS), reproduced with permission (21, 60, 61).

EEfRT (19): The EEfRT is a computer test assessing effort-based decision-making as an objective measure of reduced reward ‘wanting’. It lasts 20 minutes during which participants repeatedly choose between a hard and an easy task in order to obtain varying amounts of money. Before each trial, participants are presented with the potential amount to be won for each task (easy task always 1€, hard task up to 4.20€) and the chance of receiving the money if the task is accomplished (12%, 50%, or 88%) (**Figure 1A**). After each trial, feedback is provided on task completion and reward payout. Studies suggest that the low probability condition (EEfRT_12) is specifically related to other measures of anhedonia and will thus be used for analysis (19, 62). Participants are encouraged to make a deliberate choice for each trial and are informed that they will receive their winnings after the study.

### 2.3 MRI paradigm

The SEXpect Paradigm, based on Walter et al. (20), was designed to assess anticipation and consummation of sexual stimuli (**Figure 1B**). Images were selected from the Nencki Affective Picture System (NAPS; 21, 60, 61) and included positively valanced emotional images (children, families, people doing sports) and positive erotic images, matched to the sexual orientation of the participants (nude men/women and couples during intimate actions). Images from both categories were selected to have similar valence and arousal ratings (Emotional: mean valence = 7.19, mean arousal = 5.18; Sexual: mean valence = 6.85, mean arousal = 5.2). In total, 40 images were used (20 erotic, 20 non-erotic). Half of the pictures of each category were preceded by arrows acting as cues indicating the nature of the following image. Arrows pointing upwards indicated an emotional and downwards an erotic image. After every image, participants rated their arousal [calm (0) – highly aroused (100)], image valence [pleasant (0) – unpleasant (100)], and sexual arousal [not sexually aroused (0) – highly sexually aroused (100)] using a remote control operated with their index fingers and thumb. While there was no time limit imposed on the rating, participants were encouraged beforehand to answer intuitively. Participants were instructed on the association between the cue and the image before going into the scanner.

### 2.4 fMRI imaging procedure and preprocessing

MRI scan was performed with a 3.0-T Siemens MAGNETOM Skyra scanner (Siemens Healthineers, Erlangen, Germany) with a 64-channel head coil. T1-weighted image was acquired with 1x1x1mm voxels and 176 slices (TR = 5000ms, TE = 2.98ms, field of view = 208x208x156mm, flip angle = 0°). Functional images during SEXpect were acquired using a T2*-weighted simultaneous multislice echo planar imaging sensitive sequence (voxel size = 2x2x2mm, number of slices = 78, TR = 1310ms, TE = 36ms, field of view = 208x208x156mm, flip angle = 64°, acceleration factor = 6). Images were smoothed with an 8mm full-width-half-maximum gaussian smoothing kernel. For data pre-processing and analysis, Statistical Parametric Mapping 12 software (SPM12; 22) was used. Pre-processing steps included slice time correction, realignment, co-registration with and segmentation of T1-images, normalization, and smoothing. In first-level analyses, contrasts were computed for the sexual anticipation vs. implicit baseline (‘Cue Sex > baseline’), for sexual anticipation vs. emotional anticipation (‘Cue Sex > Cue Emotion’), for sexual consummation vs. implicit baseline (‘Stimulus Sex > baseline’), and for sexual vs. emotional consummation (‘Stimulus Sex > Stimulus Emotion’).

### 2.5 Data processing and statistical analyses

Statistical tests were performed using SPSS 29 (23). Clinical scores were compared between groups by two-sample t-test or Mann-Whitney-U, differences in distribution by Chi-square or Fisher’s-Exact test. EEfRT performance and image ratings during the SEXpect were analysed using repeated measures ANOVA (EEfRT: 2x3 – Group (SZG, HC) x Chance (12%, 50%, 88%), SEXpect: 2x2x2 – Group (SZG, HC) x Stimulus (sexual image, emotional image) x Cue (cued, uncued).

#### 2.5.1 Whole-brain analysis

Second-level analyses were performed with the participants’ sex as a covariate. Differences in blood-oxygen-level dependent (BOLD) response between SZG and HC were compared with two-sample t-test in all four conditions (‘Cue Sex > baseline’, ‘Cue Sex > Cue Emotion’, ‘Stimulus Sex > baseline’, ‘Stimulus Sex > Stimulus Emotion’). A p <.05 using FWE correction on a cluster-level and a voxelwise T-threshold of 3.25 was deemed statistically significant. As this study is the first to examine neural correlates of SF deficits in SZ, it was deemed valuable to lower the T-threshold to 2.4, if the initial analyses yielded no results to avoid missing potentially informative results. Beta-weights were extracted from regions with significant group differences for subsequent analysis with marsbar toolbox (24).

#### 2.5.2 Clinical data and brain activations

Associations between clinical scores and extracted beta-weights of areas from the whole-brain analysis were tested using Spearman’s rho, for non-parametric variables, or Pearson correlation, for parametric variables. Hence, prolactinergic potential of the medication and brain activations were correlated using Spearman and all other clinical variables (ASEX, CSFQ, DARS, EEfRT_12, PANSS Neg, CPZ dose, time of taking medication) using Pearson. Participants were excluded if brain activity (beta weight) or EEfRT performance showed absolute z-scores of 2.5 or greater. Deviations in self-report questionnaires were assumed to reflect true manifestation of the symptom and were not used as ground for exclusion. Bonferroni correction was applied to all correlational analyses for each brain region.

#### 2.5.3 Exploratory analyses

As group comparisons for the contrast ‘Cue Sex > Cue Emotion’ yielded few results, we split the paradigm into 1^st^ and 2^nd^ half *post hoc* to rule out a delayed learning of cue – image contingencies in SZG being responsible for the small effects. The assumption was based on findings of deficient WM and positive reinforcement learning in SZ (13, 25, 26). We exploratorily correlated CSFQ subscores and DARS social reward sensitivity subscale with BOLD responses to investigate potentially relevant aspects of anhedonia and SF deficits.

## 3 Results

### 3.1 Clinical characteristics

The sample comprised 29 SZG and 29 HC, with groups matched in gender, age, handedness, and academic/vocational education but more HC were currently in a romantic partnership compared to SZG (**Table 1**). SZG time since onset was 11.38 years (SD = 10) and comorbid diagnoses were present in 58.6% of patients; Most commonly neurotic/somatoform (N = 10; 34.48%) and affective (N = 8; 27.59%) disorders, followed by attention deficit disorder (N = 5; 17.24%), substance use disorders (N = 4; 13.79%), and personality disorders (N = 4; 13.79%). Cumulative time of taking antipsychotic medication was 10.59 years (SD = 9.54), current CPZ dose 419.86mg (SD = 446.85), and 16 (58.5%) patients were currently taking medication with low to medium prolactinergic potential, prescribed antipsychotic classes are listed in **Table 1**, specific agents are reported in the **Supplementary Materials S1**. SZG displayed more severe negative symptoms (T(30.47) = 7.585, p <.001, *d* = 1.99), lower hedonic capacity (T(55) = -2.681, p = .005, *d* = -.71), and worse sexual functioning (*CSFQ*: T(43.34) = -2.94, p = .003, *d* = -.797; *ASEX*: T(45.62) = 3.3, p <.001, *d* = .866) than HC (**Table 1**). According to CSFQ and ASEX, 11 SZG (37.93%)/18 SZG (62.07%) and 2 HC (6.9%)/7 HC (24.14%) reached the cut-off for SD classification (*CSFQ:* χ² (1) = 8.99, p = .003, V = .401; *ASEX:* χ² (1) = 8.51, p = .007, V = .383). There was no significant difference between participants who were romantically involved (N = 34) vs. those who were not (N = 24) regarding SF scores (*CSFQ*: T(54) = -.059, p = 477; *ASEX*: T(56) = -.556, p = .29). Within SZG, there was likewise no difference between these groups (involved: N = 10, not involved: N = 19) (*CSFQ*: T(10.184) = -1.192, p = .13; *ASEX*: T(27) = 1.21, p = .118). Further, SZG showed higher positive symptom and general psychopathology scores (**Table 1**). In the EEfRT, a group difference (F(1) = 4.105, p = .048, η²p = .069), as well as, a main effect of chance of winning (F(2) = 67.659, p <.001, η²p = .552) and an interaction effect between group and chance (F(2) = 4.659, p = .011, η²p = .078) emerged (descriptives in **Table 1**). Overall, HC chose the hard task more often than SZG and hard tasks were chosen more often in medium (50%) and high (88%) probability trials compared to low probability trials by both groups. In the healthy control group, the number of hard tasks increased more steeply from the low/medium probability trials to the high probability trial compared to SZG.

**Table 1:** Demographic information, clinical data, and performance on EEfRT.

Variable	M(SD)/Frequency	Goup		T(df)/χ²/rho	p	*d/V*
		SZG	HC			
Demographic information						
Age	M (SD)	35.59 (10.61)	32.45 (12.47)	1.032 (54.597)	.306	-.271
Gender (m/f)	N	18/11	18/11	.00	1	
Handedness (r/l)	N	23/6	23/6	.00	1	
Academic Education (none/lower secondary/higher secondary)*	N	1/16/12	0/17/12	.014	.913	
Vocational Education (none/vocational training/Bachelors/Masters/PhD)	N	14/9/4/1/1	9/15/1/3/1	.116	.345	
Relationship Status (yes/no)	N	10/19	24/5	14.007 (2)	**<.001**	**.491**
Hormonal Medication (e.g. birth control) (yes/no)	N	0/29	4/25	4.296 (1)	.056	.272
SSRIs/SNRIs/Benzodiazepines (yes/no)	N	11/18	0/29	13.574 (1)	**<.001**	**.484**
Antipsychotic generation (first/second/both)	N	0/25/3	0/0/0			
Group differences on PANSS scales						
PANSS Neg	M (SD)	14.93 (4.84)	7.97 (1.01)	7.585 (30.47)	**<.001**	**1.99**
PANSS Pos	M (SD)	15.31 (4.8)	8 (1.2)	7.96 (31.46)	**<.001**	**1.442**
PANSS Psychopath	M (SD)	32.97 (6.801)	19.69 (3.465)	9.37 (41.62)	**<.001**	**1.767**
Group differences on anhedonia and sexual functioning						
DARS	M (SD)	76.24 (15.93)	86.13 (11.68)	-2.681 (55)	**.005**	**-.71**
CSFQ	M (SD)	47.28 (9.12)	53.31 (5.76)	-2.94 (43.34)	**.003**	**-.797**
ASEX	M (SD)	17.27 (4.34)	14.1 (2.81)	3.3 (45.62)	**<.001**	**.866**
EEfRT performance descriptives						
EEfRT_12	M (SD)	12.83 (14.39)	12.57 (16.77)			
EEfRT_50	M (SD)	27.39 (23.17)	38.47 (28.94)			
EEfRT_88	M (SD)	41.52 (33.14)	61.62 (22.97)			

*according to the German educational system: 9 years = basic education, 10 years = Mittlere Reife, 11 years = Fachhochschulreife, 12 to 13 years = A-Levels.

EEfRT_XX, percentage of hard task choices at 12%, 50%, or 88% chance of winning.Bold values indicate statistical significance at p ≤ .05.

#### 3.1.1 Clinical intercorrelations and medication effect

Measures of SF correlated significantly with negative symptoms and the DARS score across the samples. Within SZG, the correlation between the CSFQ and DARS remained significant, but did not survive Bonferroni correction (**Table 2**). Within SZG, binary logistic regression analyses of PANSS Neg, DARS, and EEfRT_12 on the classification of SD according to the CSFQ and ASEX showed a significant effect of hard task choices during EEfRT on the chance of SD classification according to the ASEX (B = -.123, SE = .055, p = .025, OR = .884). The model was significant (χ²(3) = 8.218, p = .042). There were no significant associations between medication parameters (time of taking antipsychotic medication, CPZ dose, prolactinergic potential) and clinical measures.

**Table 2:** Correlations between the clinical parameters across samples and within SZG.

Sexual Functioning Score	PANSS Neg			DARS			EEfRT_12		
	r (r^2^)	p	CI	r (r^2^)	p	CI	r (r^2^)	p	CI
Across samples									
CSFQ Score	-.367(.135)	.005*	[-.575; -.115]	.361(.13)	.007*	[.106;.571]	.242(.059)	.081	[-.03;.481]
ASEX Score	.394(.155)	.002*	[.152;.592]	-.304(.092)	**.022**	[-.523; -.047]	-.134(.018)	.328	[-.386;.136]
Within SZG									
CSFQ Score	-.172(.03)	.392	[-.518;.223]	.462(.213)	**.018**	[.091;.72]	.283(.08)	.17	[-.126;.61]
ASEX Score	.185(.034)	.338	[-.195;.516]	-.273(.075)	.16	[-.586;.111]	-.283(.08)	.152	[-.599;.108]

*significant after Bonferroni (threshold = p = .05/3 = .017).Bold values indicate statistical significance at p ≤ .05.

### 3.2 Behavioral data

**Figure 2:**
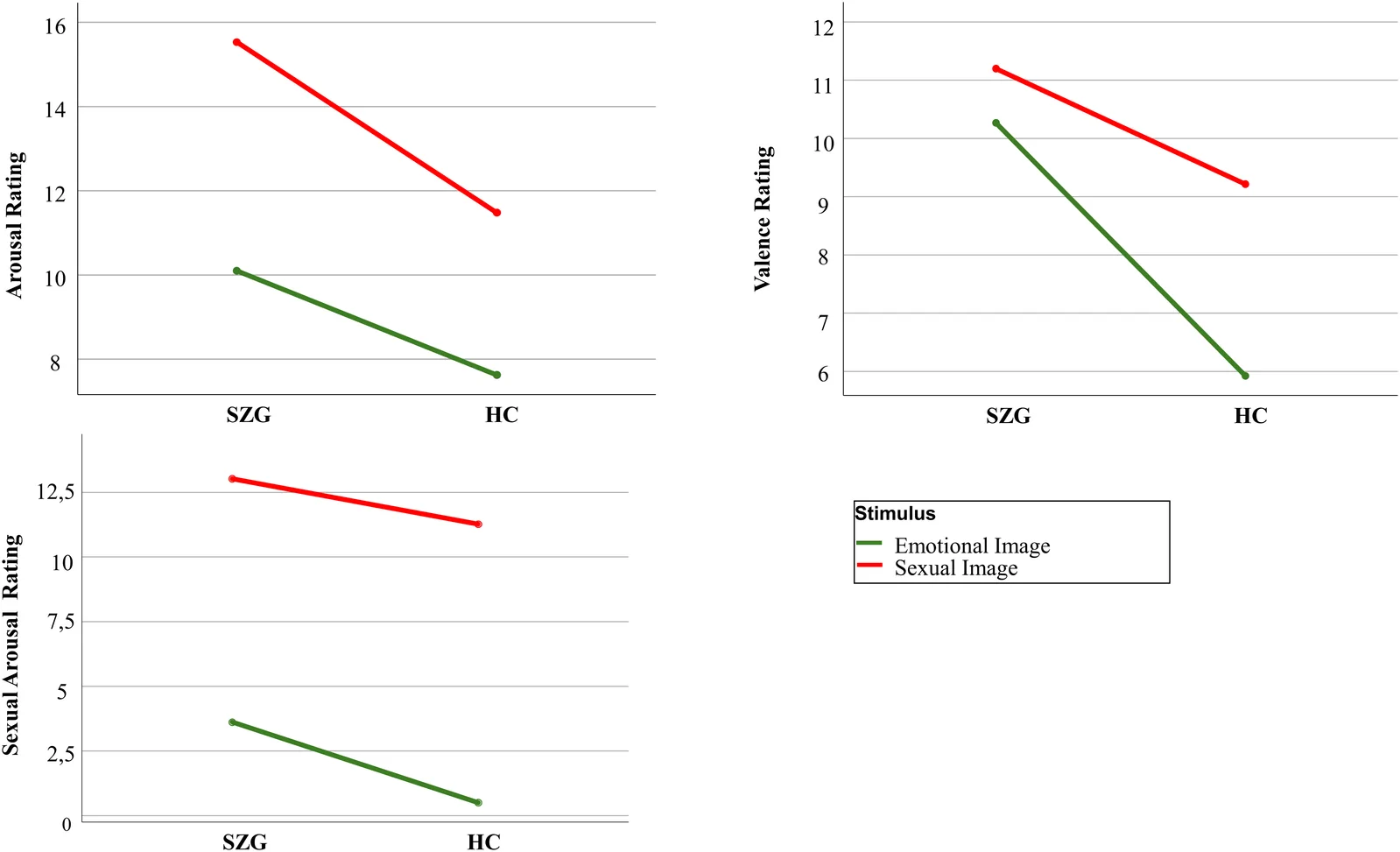
Repeated measures ANOVA of SEXpect image ratings (2x2x2 – group (HC, SZG) x stimulus (sex, emotion) x cue (cued, uncued). For (sexual) arousal higher values indicate greater arousal, for valence higher values indicate a less pleasant experience.

Arousal ratings differed significantly between groups, as SZG reported higher arousal than HC after both image categories (SZ: 12.82(6.5), HC: 9.55(5.99), F(1) = 6.191, p = .016, η²p = .1). Arousal ratings additionally exhibited a significant effect of stimulus type with higher arousal ratings after sexual than after emotional images (Sex.: 13.5(7.25), Emo.: 8.86(5.63), F(1,56) = 41.258, p <.001, η²p = .424). Valence ratings likewise exhibited main effects of ‘Group’ and ‘Stimulus’. SZG indicated lower valence than HC after both image categories (SZ: 10.73(6.57), HC: 7.57(4.73), F(1) = 6.389, p = .014, η²p = .102) and valence ratings after sexual images were more negative as opposed to after emotional images (Sex.: 10.21(6.68), Emo.: 8.09(5.24), F(1,56) = 8.238, p = .006, η²p = .128). Lastly, sexual arousal ratings only showed a significant difference between image categories with sexual images being rated as more sexually arousing than emotional images (Sex.: 12.14(7.9), Emo.: 2.06(5.15), F(1,56) = 107.475, p <.001, η²p = .657). Effects are displayed in **Figure 2**.

### 3.3 Whole-brain analyses

**Figure 3:**
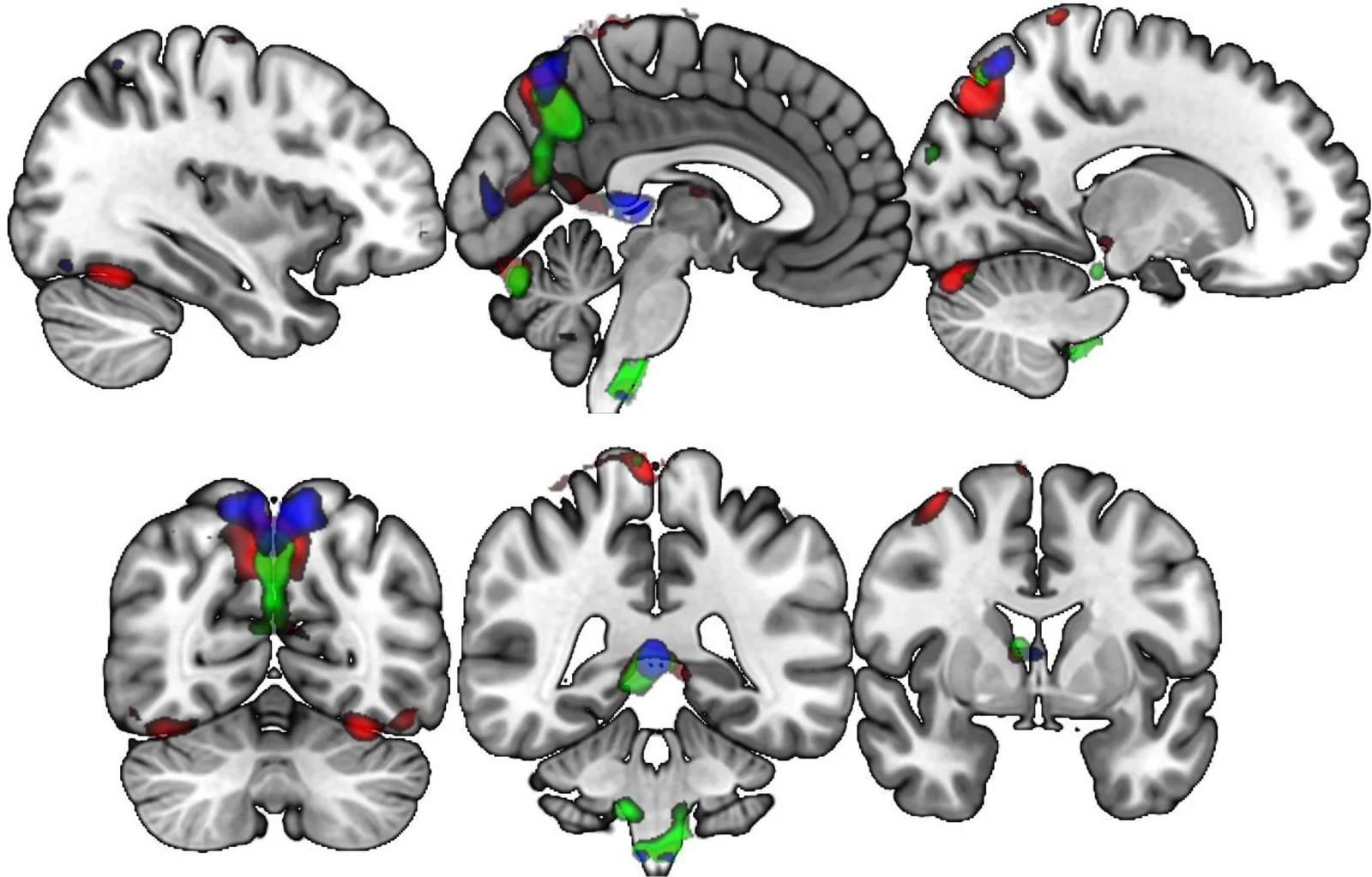
Whole-brain analysis of SEXpect group comparison (HC > SZG). Red: ‘Cue Sex > baseline’; Green: ‘Cue sex > Cue Emotion’; Blue: ‘Stimulus Sex > baseline’. Specification: significant at FWE cluster-level p <.05, T-threshold = 3.25 (‘Cue Sex > baseline’; ‘Stimulus Sex > baseline’), significant at FWE cluster-level p <.05, T-threshold = 2.4 (‘Cue Sex > Cue Emotion’) Sagittal: x = -55, -3, 15; Coronal: y = -65, -40, 2.

Expectedly, sexual stimuli caused increased activation in temporal and occipital areas compared to emotional images. During ‘Cue Sex > baseline’, HC showed higher response compared to SZG in four major clusters with peaks in the precuneus, calcarine gyrus, paracentral lobule and lingual gyrus. Initial group comparison at p_uncorr._ = .001 and p_FWE cluster_ = .05 for ‘Cue Sex > Cue Emotion’ yielded no results, but a more exploratory threshold of p_uncorr._ = .01 revealed less activity in the precuneus and cerebellum in SZG compared to HC. Further, HC showed widespread increases in brain response for ‘Cue Sex > Cue Emotion’, while in SZG no significant differentiation of cues was apparent. For the consumption condition (‘Stimulus Sex > baseline’), there was a differences with HC showing more activation in the calcarine gyrus than SZG. For ‘Stimulus Sex > Stimulus Emotion’, no group differences were found. All effects of SEXpect analyses are displayed in **Table 3**, **Figure 3**.

**Table 3:** Whole-brain analysis of the SEXpect contrasts; between and within group contrasts.

Constrast	Peak coordinates			Tpeak	pFWE	k(E)	Anatomic region
	x	y	z				
‘Stimulus Sex > Stimulus Emotion’							
HC *	-40	-58	-4	7.87	.000	10005	L Mid. Temporal Gyrus, L Inf. Occipital Gyrus, L Mid. Occipital Gyrus
	38	-28	-6	7.6	.000	13209	R Hippocampus, R Mid. Occipital Gyrus, R Inf. Occipital Gyrus
SZG *	48	-68	0	6.11	.000	2041	R Mid. Temporal Gyrus, R Inf. Temporal Gyrus
	-46	-60	0	6.03	.000	2342	L Mid. Temporal Gyrus, L inf. Temporal Gyrus, Medial OFC
	6	28	-2	4.44	.032	331	Medial OFC
‘Stimulus Sex > baseline’							
HC > SZG *	34	-56	8	5.09	.000	1212	Calcarine Gyrus
‘Cue Sex > Cue Emotion							
HC*	-20	-34	-50	5.37	.001	647	L Cerebellum_X, L Uvula, Vermis 9
	24	-82	24	5.19	.000	719	R Sup. Occipital Gyrus, R Sup. Parietal Cortex
	-2	-24	-14	5.14	.000	1327	VTA, R Hippocampus
	-36	-76	-20	4.59	.003	538	L Mid. Occipital Gyrus
	10	48	22	4.53	.000	1445	preACC
	44	-72	26	4.4	.01	409	R Mid. Occipital Gyrus, R Mid. Temporal Gyrus
HC > SZG **	-2	-58	38	4.06	.000	2674	L Precuneus, L Calcarine Gyrus, R Cuneus
	14	-44	-48	3.98	.001	1947	Bilateral Uvula, L Cerebellum_VIII
‘Cue Sex > baseline’							
HC > SZG *	10	-72	44	5.13	.000	1140	Bilateral Precuneus, L MCC
	-16	-62	14	4.96	.000	1413	L Calcarine Gyrus, R Sup. Occipital Gyrus, L Precuneus
	0	-32	72	4.62	.003	513	Bilateral Paracentral Lobule
	22	-72	-10	3.89	.05	269	R Lingual Gyrus, R Fusiform Gyrus, R Cerebellum_VI

*significant at FWE cluster-level p <.05, T-threshold = 3.25, **significant at FWE cluster-level p <.05, T-threshold = 2.4.

### 3.4 Functional brain activity and clinical parameters

Correlations between anticipatory and consummatory brain response and clinical measures of sexual function, anhedonia and negative symptoms across the samples showed several significant associations, especially negative symptoms were associated with all clusters (**Table 4**). Within SZG, only the association between negative symptoms and activation in the paracentral cluster (r = -.382, r^2^ = .15, p = .041, CI: [-.657; -.018]) during ‘Cue Sex > baseline’ and the cerebellar cluster during ‘Cue Sex > Cue Emotion’ (r = -.449, r^2^ = .2, p = .015, CI: [-.7; -.098]) remained, neither survived Bonferroni (all effects in **Supplementary Material S2**). Medication parameters showed no significant association with brain responses during SEXpect.

**Table 4:** Correlations between anticipatory and consummatory BOLD response and clinical parameters across the samples.

Cluster	CSFQ			ASEX			DARS			PANSS Neg			EEfRT_12		
	r(r^2^)	p	CI	r(r^2^)	p	CI	r(r^2^)	p	CI	r(r^2^)	p	CI	r(r^2^)	p	CI
’Cue Sex > baseline‘															
Precuneus Cluster	.294(.086)	.269	[.028; .521]	.152(.023)	.437	[-.118;.401]	.155(.024)	.254	[-.112;.402]	-.364(.132)	.005*	[-.57; -.114]	.294(.086)	**.031**	[.028; .521]
Calcarine Cluster	.207(.043)	.127	[-.06; .445]	-.216(.047)	.104	[-.449;.045]	.237(.056)	.076	[-.026;.468]	-.468(.219)	<.001*	[-.648;-.238]	.211(.045)	.123	[-.058; .451]
Paracentral Cluster	.298(.089)	**.026**	[.038; .52]	-.249(.062)	.059	[-.477; .01]	.221(.049)	.098	[-.042;.456]	-.567(.321)	<.001*	[-.72;-.362]	.231(.053)	.09	[-.037; .467]
Lingual Cluster	.299(.089)	**.027**	[.036;.523]	-.132(.017)	.326	[-.38;.133]	.418(.175)	.001*	[.174;.613]	-.437(.191)	<.001*	[-.626;-.199]	.198(.039)	.152	[-.074; .442]
’Cue Sex > Cue Emotion‘															
Precuneus Cluster	.163(.027)	.23	[-.104;408]	-.199(.04)	.135	[-.435;063]	.209(.044)	.119	[-.055;.445]	-.471(.222)	<.001*	[-.65;-.242]	.185(.034)	.177	[-.085; .429]
Cerebellum Cluster	.202(.041)	.14	[-.067;443]	-.204(.042)	.128	[-.441;.06]	.193(.037)	.154	[-.074;.434]	-.507(.257)	<.001*	[-.678;-284]	.104(.011)	.454	[-.168; .362]
’Stimulus Sex > baseline‘															
Calcarine Cluster	.472(.223)	<.001*	[.239; 654]	-.323(.104)	**.013**	[-.537;-.071]	.333(.111)	**.011**	[.08;.546]	-.499(.249)	<.001*	[-.671;-.277]	.253(.064)	.062	[-.013; .486]

*significant after Bonferroni (threshold = p = .05/5 = .01).Bold values indicate statistical significance at p ≤ .05.

### 3.5 Exploratory analyses

#### 3.5.1 Subcomponents sexual dysfunction & anhedonia

CSFQ subscales and the DARS social reward subscale were exploratorily used in a repetition of the analyses above to reveal correlates of specific sexual functions and social anhedonia. All scales, except CSFQ ‘Desire/Interest’, showed significant group differences and were partly correlated with PANSS Neg (**Supplementary Materials S3**, **S4**). Across the samples, anticipatory and consummatory BOLD responses correlated significantly with CSFQ subscales and DARS social scale (**Table 5**). Within SZG, associations between the ‘Cue Sex > baseline’ response in the lingual cluster and sexual pleasure (r = .407, r^2^ = .17, p = .035, CI: [.032;.682]) remained significant but did not survive Bonferroni correction (all effects in **Supplementary Material S5**).

**Table 5:** Correlations between anticipatory and consummatory BOLD response and CSFQ and DARS subscales across the samples.

Cluster	Pleasure			Desire					Interest						
	r(r^2^)	p	CI	r(r^2^)		p		CI	r(r^2^)		p			CI	
‘Cue Sex > baseline’															
Precuneus Cluster	.398(.158)	.003*	[.149; .6]	.3(.09)		**.026**		[.038; .524]	-.007(.0)			.958			[-.272; .258]
Calcarine Cluster	.379(.144)	.004*	[.129; .584]	.313(.098)		**.019**		[.055; .532]	.043(.002)			.754			[-.223; .302]
Paracentral Cluster	.417(.174)	.001*	[.173; .613]	.402(.162)		.002*		[.155; .601]	.11(.012)			.42			[-.158; .362]
Lingual Cluster	.307(.094)	**.023**	[.045; .529]	.351(.123)		**.009**		[.095; .564]	.036(.001)			.791			[-.231; .299]
‘Cue Sex > Cue Emotion’															
Precuneus Cluster	.333(.111)	**.012**	[.077; .548]	.169(.029)	.214		[-.099; .413]		.031(.001)			.821			[-.234; .291]
Cerebellum Cluster	.295(.087)	**.029**	[.032; .52]	.227(.052)	.096		[-.041; .464]		.011(.0)			.937			[-.255; .275]
‘Stimulus Sex > baseline’															
Calcarine Cluster	.503(.253)	<.001*	[.276; .676]	.471(.222)		<.001*		[.237; .653]	.303(.092)			**.023**			[.044; .524]
	Arousal			Orgasm					Social Reward						
	r(r^2^)	p	CI	r(r^2^)		p		CI	r(r^2^)		p			CI	
‘Cue Sex > baseline’															
Precuneus Cluster	.12(.014)	.383	[-.15; .373]	.031(.001)		.82		[-.236; .294]	.216(.047)		.107			[-.048; .451]	
Calcarine Cluster	.177(.031)	.193	[-.09; .42]	.106(.011)		.437		[-.161; .359]	.292(.085)		**.026**			[.036; .511]	
Paracentral Cluster	.282(.08)	**.035**	[.02; .507]	.126(.016)		.354		[-.141; .377]	.318(.101)		**.015**			[.065; .532]	
Lingual Cluster	.201(.04)	.142	[-.068; .442]	.204(.042)		.134		[-.064; .446]	.406(.165)		<.001*			[.227; .644]	
‘Cue Sex > Cue Emotion’															
Precuneus Cluster	.168(.028)	.217	[-.1; .412]	-.013(.0)		.924		[-.275; .251]	.343(.118)		.008*			[.093; .552]	
Cerebellum Cluster	.125(.016)	.361	[-.145; .378]	.119(.014)		.387		[-.151, .372]	.342(.117)		**.009**			[.089; .553]	
‘Stimulus Sex > baseline’															
Calcarine Cluster	.339(.115)	**.011**	[.083; .552]	.309(.095)		**.02**		[.05; .529]	.384(.147)		.003*			[.14; .584]	

*significant after Bonferroni (threshold = p = .05/5 = .01).Bold values indicate statistical significance at p ≤ .05.

#### 3.5.2 Analysis of a learning effect during the SEXpect

**Figure 4:**
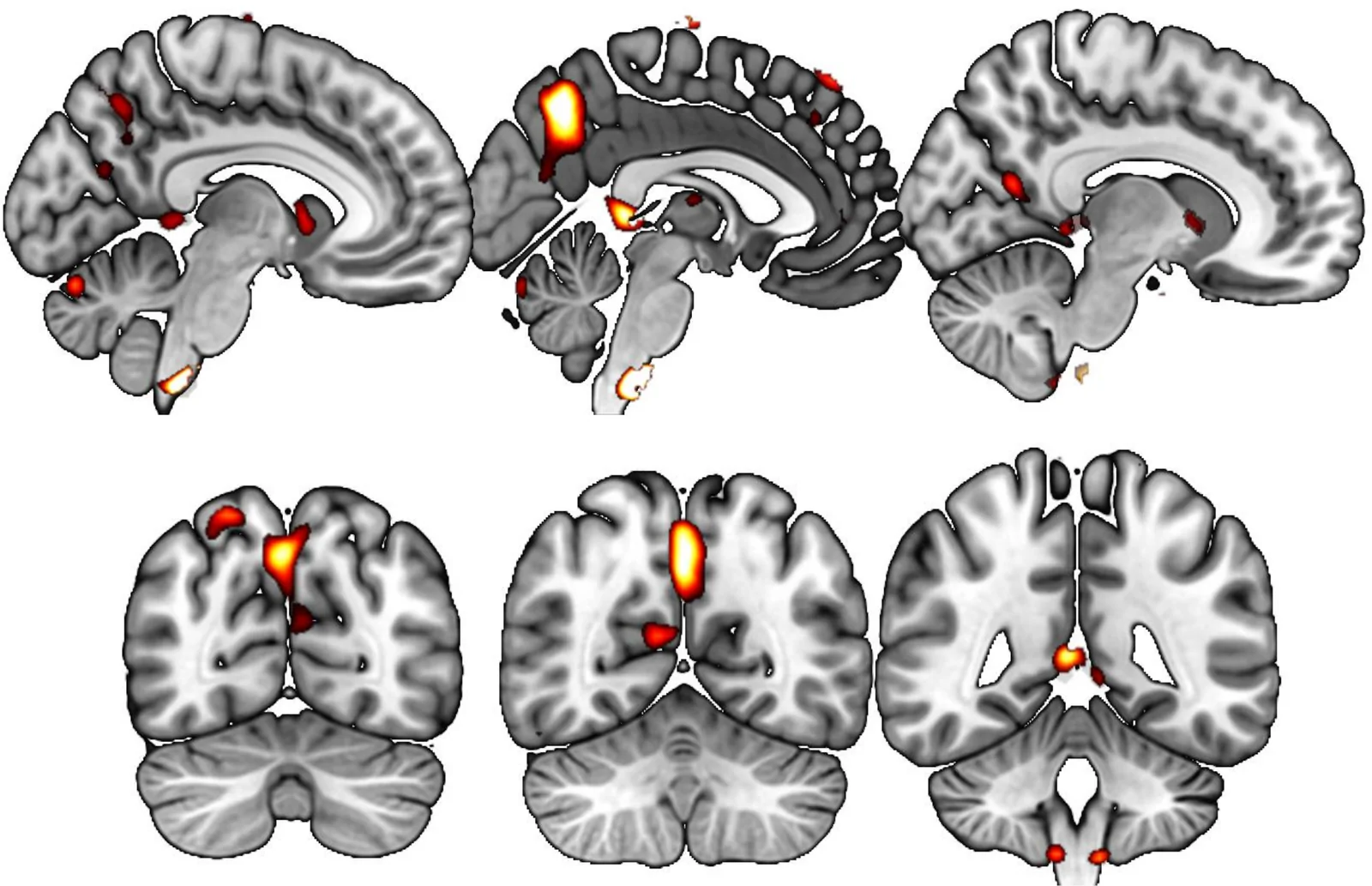
Group comparison (HC > SZG) in the contrast ‘Cue Sex > Cue Emotion 2^nd^ Half’ of the SEXpect. Specification: significant at FWE cluster-level p <.05, T-threshold = 3.25, Sagittal: x = -7, -2, 10; Coronal: y = -70, -60, -45.

The main contrast for the learning effect (‘Cue Sex > Cue Emotion 2nd Half’ > ‘Cue Sex > Cue Emotion 1st Half’) showed no significant group differences. In the 2^nd^ half of the paradigm, HC displayed significantly higher activation for ‘Cue Sex > Cue Emotion’ in the calcarine gyrus compared to SZG, while in the 1^st^ half no group differences were detected (**Figure 4**; detailed listing of effects in **Supplementary Material S6**).

## 4 Discussion

To our knowledge, this is the first study to investigate neurofunctional mechanisms underlying SF in SZ regarding anhedonia and sexual reward processing. We mainly found aberrant brain activations during anticipation, rather than during consummation, and saw associations with negative symptoms, anhedonia and aspects of sexual functioning across reward processing stages. SZG showed reduced functional brain response during anticipation in the bilateral precuneus and several occipital and cerebellar regions. Interestingly, HC showed clear differentiating brain responses between the sexual and non-sexual cue, which was not observed in the SZ group. Across the samples, the anticipatory brain responses correlated consistently with negative symptoms and partly with aspects of anhedonia and sexual functioning. When presented with sexual images, the only group difference was a reduced activation in the calcarine gyrus in SZG and there were no significant group differences for the specific brain response to sexual vs. non-sexual images. Across the samples, consummatory brain activity in the calcarine cluster correlated with all clinical scales, except EEfRT performance. This study was based on the assumption that SD in SZ is not a stand-alone phenomenon caused mainly by antipsychotic medication, as often implied in the literature and by health care professionals (5, 8), but that it is rather linked to the negative symptomatology, specifically anhedonia. The present results match previous findings of high prevalence of SD in SZ (4), however, it should be noted that there was a significant difference between the groups regarding current romantic relationships, as most HC were currently with a partner and most SZG were single. In the present study, we did not see differences in SF scores between single and romantically involved patients and the questionnaires also refer to masturbation as sexual activity and include items assessing general liking of sexual material, as to minimize the effect of an available partner. Nevertheless, it cannot be ruled out that relationship status is a contributing factor. This would be in line with Edinoff et al. (8) reporting higher likelihood of SD in unmarried, divorced, or widowed patients and the understanding of SF deficits in this population to be a secondary symptom due to everyday struggles (5). As such, future studies may consider integrating this aspect when investigating the nature of SF deficits in SZ. We did find strong correlations between measures of anhedonia, negative symptoms, and SF across the two groups, suggesting a relationship between these phenomena. More importantly, while correlational analyses within the SZ group showed no robust associations between clinical measures, a logistic regression in the group revealed that EEfRT performance was a significant predictor for SD classification according to the ASEX, indicating a meaningful association within this psychiatric population. It should be noted that the EEfRT captures motivational deficits and may not fully encompass hedonic capacity per se. However, the previous literature highlights the close association between decreased motivation and anticipatory anhedonia in particular. Anhedonia questionnaires have repeatedly shown strong correlations with EEfRT performance (e.g.: 19, 27, 62) and, as Rømer Thomsen (28) pointed out, pursuing a reward involves both the ability to understand reward cues as motivational signals, motivation to pursue, and effort computation. The author additionally states that behavioural measures may uncover aspects of anhedonia that present outside of the conscious experience. As such, we argue that the deficits captured by the EEfRT served as a good representation for the hedonic and reward processing capacity of the participants, especially for the anticipatory phase. Medication parameters did not correlate with any of the clinical scales, lending cautious support to the assumption that medication may not be the sole contributor to SF deficits in SZ. However, the medication parameters assessed in the present study likely did not capture the full scope of pharmacological impact on clinical presentations, which should be kept in mind when interpreting this result.

Regarding functional brain response, most group differences were seen during anticipation and not consumption, which fits with other accounts of consummatory pleasure being largely intact in SZ (10). It has to be noted, that SZG generally rated viewing images as less pleasant compared to HC. Thus, the observed anticipatory deficits may not reflect an impairment of these processes themselves, but rather that the rewarding stimuli were not perceived as such by this group and therefore not anticipated when the cue was presented. However, the reported valence by SZG did not differ between emotional and sexual images, so the discomfort may have arisen due to a general tension during the scanning and not due to reduced liking of the sexual images per se. Furthermore, on a neurofunctional level, SZG showed similar consummatory brain activation patterns to HC. The only notable difference was a hypoactivation of the calcarine gyrus in SZG, when presented with sexual images compared to baseline. Occipital regions have been shown to respond stronger to visual stimuli if they have motivational or affective value for the subject (29), which accords with Walter et al. (20) reports of stronger occipital response to erotic compared to neutral or emotional images. This pattern was observed for the contrast ‘Sexual Stimulus > Emotional Stimulus’ in HC but not SZG, however, this difference did not reach statistical significance. There is ample evidence for structural and functional alterations of the occipital cortex in SZ (30) and previous studies find hypoactivations in the fusiform, calcarine and lingual gyri along with other occipital regions during reward learning and anticipation and social affective processing in SZ (31–33). The reduced consummatory response in the calcarine gyrus in SZG indicates a generally blunted response to visual stimuli in SZ, but not necessarily a specific devaluation of sexual content. It is also conceivable that the reduced occipital response is not representative of deficient reward processes but rather an underlying limitation in the cognitive processing of visual stimuli per se. This would be in line with previous accounts of reduced visual processing in SZ, which could impact other cognitive processes downstream (34, 35). As both image types were social in nature, it is possible that people with SZ generally respond less to social and emotional stimuli. Across the samples, we found correlations between the BOLD response in the calcarine gyrus and SF and negative symptoms, with greater calcarine response indicating better functioning. The exploratory analyses further indicated an association of calcarine response with sexual pleasure and desire and social reward sensitivity. Within the SZ group alone, none of these associations remained significant, however. Currently, little is known about clinical correlates of occipital dysfunction in SZ, except its association with visual hallucinations (30). Bjorkquist & Herbener (31) mentioned medium sized effects of correlations between reduced occipital activity in response to affective social vs. non-social images and PANSS scales. Concludingly, patients seem to experience visual sexual rewards similarly to healthy controls. Nevertheless, some deviations were found that indicate reduced valuation of images with social components, which could potentially contribute to negative symptoms and SD and may in part drive the neural anticipation deficits as well.

During the simple anticipatory contras (‘Cue Sex > baseline’), group differences in BOLD response were observed in the precuneus, several occipital regions, and the paracentral lobule. For the comparative contrast ‘Cue Sex > Cue Emotion’, group differences only emerged at the lower, more exploratory, threshold in the precuneus and vermal uvula. In all areas, HC showed greater activations compared to SZG. The previously described value-dependent activation of occipital regions suggests that the blunted response to reward cues reflects the lack of value attributed to them or, as mentioned above, that the visual processing of the cues is likewise impaired. The precuneus is involved in multiple cognitive functions, including updating WM and processing rewards, due to its high degree of connectivity (36). There is evidence for its involvement in reward anticipation (37, 38) and the processing of sexual stimuli (20, 39, 40). Its structural and functional impairments in SZ have been indicated as potential reasons for deficits in emotional processing and symptoms like apathy (41). The cerebellum plays a role in reward consumption and prediction and general hedonic processes. Of particular interest are the cerebellar lobule VI and vermal uvula. The former showed group differences during ‘Cue Sex > baseline’ and has been implicated in the salience network, coding attentional shifts and reactions to important cues (42, 43). The latter, the vermal uvula, showed group differences during ‘Cue Sex > Cue Emotion’ at the exploratory threshold. Previous studies reported it to be involved in reward anticipation (42, 43) and to respond stronger to sexual than non-sexual images (44–46). Grey matter volume reductions and altered functional activity in cerebellar regions are also consistently reported and associated with cognitive abilities and symptoms in SZ (47, 48) and multiple studies find reduced response in the cerebellar cortex during reward anticipation (32, 49, 50). While the effects in the uvula, specifically, are not surprising given the findings from previous studies, the present results are exploratory and thus should be interpreted with caution regarding a potential role of the vermal uvula in SF deficits in SZ. The paracentral lobule, spanning between post and precentral gyri, is a sensorimotor region and represents lower extremities and genitalia and is involved in motor planning and execution due to connections to the supplementary motor area and primary motor cortex (51, 52). Fittingly, pre- and postcentral gyri are consistently mentioned to show increased activity when participants are presented with visual sexual stimuli (45, 46). Gao et al. (53) reported reduced spontaneous activity in the paracentral lobuli in SZ and hypoactivations in pre- and postcentral regions have been found during reward anticipation and consummation (32, 50, 54). In the present study, group differences were only seen for the simple anticipatory contrast (‘Cue Sex > baseline’) but not for the specific comparison between the cues predicting an emotional as opposed to a sexual image. This could suggest a generally blunted reaction to abstract cues and not necessarily a reduced value of the sexual cue itself and represent reduced motor preparation as cues may not be associated with anything worth obtaining. Across both groups, we found strong negative associations between the anticipatory BOLD responses (‘Cue Sex > baseline’) in all relevant clusters and negative symptoms, as well as between anhedonia and the lingual cluster. The exploratory correlation analyses further revealed positive associations between sexual pleasure and anticipatory reactions (‘Cue Sex > baseline’) in the precuneus, calcarine gyrus, and paracentral lobuli, between sexual desire and the response in the paracentral lobuli, and between social reward sensitivity and the lingual gyrus response. Within SZG, only the associations between negative symptoms and BOLD response in the paracentral lobuli (‘Cue Sex > baseline’) and the response in the cerebellum (‘Cue Sex > Cue Emotion’) remained, which did, however, not survive statistical correction. In the exploratory analyses, a positive association between the neural response to ‘Cue Sex > baseline’ in the lingual gyrus and sexual pleasure was seen but did not survive correction. The findings from the whole sample analyses are in line with reports of a relationship between precuneus volume and trait anhedonia (55) and between its functional connectivity and avolition-apathy symptoms (56). The cerebellum and its functional connectivity to other regions have been linked to multiple cognitive deficits, e.g., WM, and seem to play a central role in the emergence of negative symptoms, as multiple studies to date found a positive effect of repetitive transcranial magnetic stimulation on negative symptoms (47, 57). There is less evidence regarding occipital correlates of negative symptoms aside from Bjorkquist & Herbener (31), reporting a medium-sized correlation with negative symptoms. Likewise, the paracentral lobuli have rarely been mentioned in relation to SZ symptoms, only Gao et al. (53) reported an association with PANSS general and negative symptom scores. Collectively, there is ample evidence for an involvement of reduced anticipatory activity in the precuneus, cerebellar, paracentral, and occipital regions in the emergence of negative symptoms and anhedonia. The present results make a first step extending these findings to SF, as associations have been found with sexual pleasure and desire, specifically. This suggests that certain aspects of SF may be connected to negative symptoms, rather than the concept of SD as a whole. It remains to emphasize that the brain-behaviour associations were only robust across the entire sample and did not reach significance or survived correction within the patient sample alone. Thus, the observed interaction between neural deficits and clinical symptoms could be driven by group differences in both variables, rather than meaningful variations within SZG. Further, results for the anticipation of an emotional vs. an erotic image (‘Cue Sex > Cue Emotion’) are based on the lowered exploratory T-threshold. As such, their limited robustness and higher risk for false-positives should be considered during interpretation. Nevertheless, our findings provide a valuable first indication of a relationship between the neurofunctional processing of sexual reward anticipation and consummation and the clinical presentation of negative symptoms and SF deficits. The present results can thus serve as a starting point for further investigations.

Several correlations between neural responses and clinical measures did not survive correction, however, we want to mention some of these as exploratory results that may serve as a basis for future studies. Across the samples, the anticipatory response (‘Cue Sex > baseline’) in the paracentral and lingual clusters were positively associated with CSFQ scores, and the response in the precuneus was positively associated with EEfRT performance. Additionally, the consummatory response (‘Stimulus Sex > baseline’) in the calcarine cluster showed a negative correlation with the ASEX and a positive correlation with DARS scores. These regions may thus be a common factor underlying the negative symptom dimension of SZ and the emergence of SF issues and should be explored further.

Importantly, BOLD responses in the clusters showing group differences during sexual reward anticipation and consumption were not associated with time of taking medication, current CPZ dose, or the prolactinergic potential of the current medication. This is in line with previous studies finding little effect of medication on brain activity (e.g.: 49, 50) and supports the notion that negative symptoms and their neural grounds cannot solely be explained by medication side effects. Nevertheless, this interpretation warrants caution, as we did not assess actual blood prolactin levels or other measures of the endocrine impact of medication. As such, we cannot rule out that other medication-related and endocrine factors contributed to the observed changes in brain activation.

As the main anticipatory contrast between the groups (HC vs. SZG for ‘Cue Sex > Cue Emotion’) showed only weak effects, it was considered that the SZ group simply needed longer to establish the cue – stimulus contingency and thus statistical differences were evened out over the course of the paradigm. However, we found no group differences in cue differentiation over time and analysis of the 1^st^ and 2^nd^ half of the paradigm revealed that there were no group differences in the first half but clearly increased activations in HC in the 2^nd^ half. As such, the absence of group differences during ‘Cue Sex > Cue Emotion’ could not be explained by delayed discrimination of cues in SZG. Nevertheless, it is rather unlikely that the lack of statistically significant effects is a true reflection of similar neural mechanisms in the groups. Within-group analyses showed distinctly greater BOLD responses in the cerebellum, occipital, parietal and temporal regions, the VTA and hippocampus, and preACC in HC for the sexual cue as opposed to the non-sexual cue, corresponding to findings in the original study on the paradigm (20). This pattern of differentiation was effectively absent in SZG and thus, a power problem seems to be the more likely cause for the lack of effects.

## 5 Limitations and conclusion

Some limitations of the present study have to be considered. First, the sample size of N = 29 per group was rather small, especially considering that some analyses had to exclude participants due to large deviations from the mean or incomplete data. One the one hand, this likely led to effects failing to reach significance, on the other hand, the sample size limits both the stability and generalizability of the presented results. Additionally, the nature of the study and recruitment process introduced a selection bias leading to a SZ sample that was comparatively highly functional with low symptom scores and good education, which could have resulted in smaller differences to the control sample. At the same time, over half of the SZG presented with psychiatric comorbidities. While this is common for this specific population, their influence on SD and sexual reward processing cannot be ruled out. Anhedonia was measured with the DARS, a tool that has not yet been commonly used in SZ. The questionnaire has been shown to capture anhedonia reliably in healthy and psychiatric populations, but future studies may additionally use scales like the TEPS, which is more often used in SZ research. Further, the influence of psychopharmaceutics and other lifestyle factors on brain function and symptoms, especially sexual functioning, could only be assessed to a certain degree. While the available medication parameters were not significantly associated with neural response or clinical presentation, a potential impact of other medication-related factors, endocrine changes, comorbidities, or relational aspects cannot be ruled out at this stage.

Lastly, it cannot be ruled out that the time of the first half of the SEXpect was not enough for SZG to learn the contingencies between the cue and the reward. In future investigations it may prove useful to introduce an initial learning trial outside the scanner to investigate whether anticipatory deficits vanish with practice and more robust associations.

All in all, our findings extend the literature on reward processing in SZ and support the notion that deficits underlying the emergence of negative symptoms and anhedonia lie in the anticipation of rewarding stimuli rather than their consumption. It should be acknowledged that the absence of robust group differences in classical reward-related regions limits stronger claims about altered core reward circuitry and further studies are necessary. Further, we were able to add to studies mentioning the high prevalence of sexual problems in SZ that seem to be connected to negative symptoms and may be partly independent of antipsychotic treatment. Most importantly, the present study gives a first insight into sexual problems in SZ on a neural basis. Activation deficits during sexual processing were not only connected to negative symptoms, as expected based on previous studies, but also to aspects of sexual functioning. While these associations presented mainly only across the sample of patient and controls, they nevertheless offer a valuable first indication of a relationship between these constructs. As such, patients should be informed by clinicians that their problems may not merely be a side effect of the medication, hopefully preventing some from discontinuing their treatment. As this is the first investigation of neural underpinnings of SD in SZ, more research is warranted. Future studies may also consider other factors that could have affected both the neural alterations and clinical deficits observed to gain more holistic insight into SF deficits in SZ. As discussed, antipsychotic and antidepressant medication, comorbid diagnoses and a deficient or delayed learning, as well as other cognitive deficits, e.g. in visual processing, could act as potential confounding variables. Further, it may be valuable to incorporate parameters like stage and duration of illness or social isolation.

## Figures and Tables

**Appendix A1:**
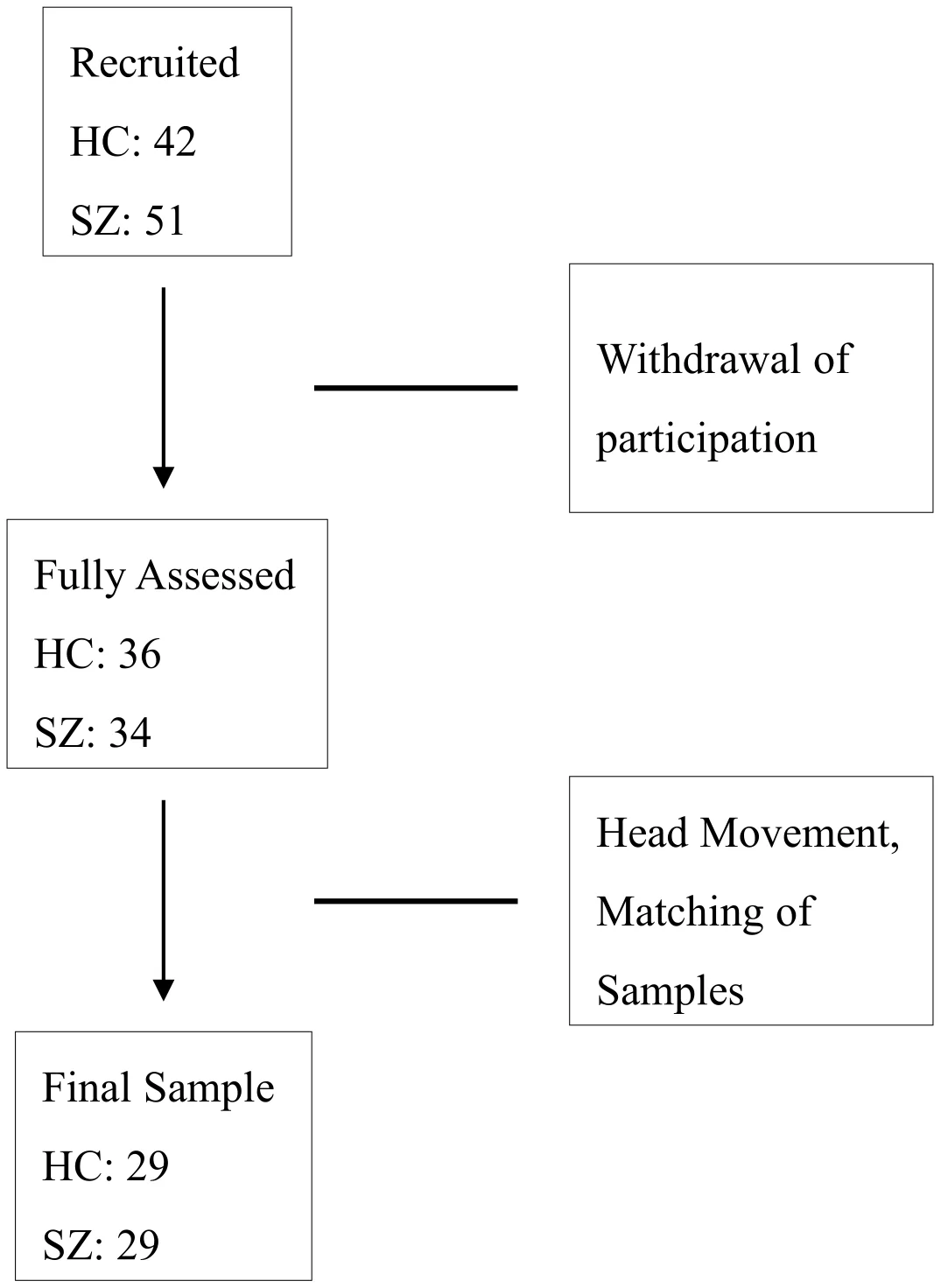
Generation of sample.

## Data Availability

The raw data supporting the conclusions of this article will be made available by the authors, without undue reservation.

## Appendix

**Appendix A2:** Classification of prolactinergic potential; according to Rahmann et al., (58) and Solmi et al. (59) – if multiple drugs were prescribed, the strongest effect was used for classification.

Drug	Category – Literature based	Category for analyses
Cariprazine (Reagila)	Neutral	0
Aripiprazole (Abilify)	Neutral	0
Quetiapine	Neutral	0
Clozapine	Neutral to slight effect	0
Amisulpride (Solian)	Strong effect	2
Pipamperone	Neutral	0
Paliperidone (Xeplion)	Strong effect	2
Risperidone (Risperidal)	Strong effect	2
Olanzapine	Medium effect	1
Melperone	Neutral to medium effect	1
Haloperidol	Strong effect	2

**Appendix A3:** DARS and CSFQ subscales.

DARS	CSFQ
**Total Score – Max. 104 points** Hobbies – Max. 36 points Food/Drink – Max. 24 points Social – Max. 24 points Sensual Experiences – Max. 20 points	**Total Score – Max. 70 points** Pleasure – Max. 5 points Desire/Frequency – Max. 10 points Desire/Interest – Max. 15 points Arousal/Excitement – Max. 15 points Orgasm/Completion – Max. 15 points

In bold - total score.
