# Supplementary material for: Diminished general and sexual hedonic capacity in schizophrenia - a clinical and neural characterization
Source: Front Psychiatry. 2026 Aug 14;17:1824523. doi: 10.3389/fpsyt.2026.1824523 (PMC13555343; doi:10.3389/fpsyt.2026.1824523)
Supplement: Supplementary file 1 [file DataSheet1.docx]

**Diminished General and Hedonic Capacity in Schizophrenia**

A Clinical and Neural Characterization

Hamers, S., Sinke C., Schultz N., Krüger THC.

**Supplementary Materials**

**S1**

Antipsychotic agents prescribed in the SZ sample

| Agent | Prescribed to N participants |
| --- | --- |
| Aripiprazole | 7 |
| Amisulpride | 4 |
| Cariprazine | 3 |
| Clozapine | 4 |
| Pipamperone | 2 |
| Risperidone | 5 |
| Haloperidol | 1 |
| Olanzapine | 3 |
| Melperone | 2 |
| Quetiapine (+ Quetiapine ret.) | 6 |
| Paliperidone | 2 |
|  |  |
| Note: most participants were prescribed multiple agents | |

**S2**

Correlations between anticipatory and consummatory BOLD response and clinical scales within SZG

**S3**

Group Differences on CSFQ and DARS subscales

**S4**

Correlations between CSFQ subscales, DARS social reward scale, PANSS negative symptom scale,

and EEfRT_12 across samples and within SZG

Note: * - significant after Bonferroni (threshold = p = .05/6 = .008)

**S5**

Correlations between anticipatory and consummatory BOLD response and clinical subscales within SZG

**S6**

Time effect in SEXpect
Within and between-group contrasts for cue differentiation in 1^st^ vs 2^nd^ half and within 2^nd^ half.

* significant at FWE cluster-level p < .05, T-threshold = 3.25, ** significant at FWE cluster-level p < .05, T-threshold = 2.4
